# Maximal Exercise Improves the Levels of Endothelial Progenitor Cells in Heart Failure Patients

**DOI:** 10.3390/cimb45030125

**Published:** 2023-02-28

**Authors:** Suiane Cavalcante, Sofia Viamonte, Rui S. Cadilha, Ilda P. Ribeiro, Ana Cristina Gonçalves, João Sousa-Venâncio, Marisol Gouveia, Manuel Teixeira, Mário Santos, José Oliveira, Fernando Ribeiro

**Affiliations:** 1 Research Centre in Physical Activity, Health and Leisure—CIAFEL, Faculty of Sports, University of Porto, 4200-450 Porto, Portugal; 2 North Rehabilitation Centre, Centro Hospitalar Vila Nova de Gaia/Espinho, 4405-565 Vila Nova de Gaia, Portugal; 3 Cytogenetics and Genomics Laboratory, Institute of Cellular and Molecular Biology, Faculty of Medicine, University of Coimbra, 3004-531 Coimbra, Portugal; 4 Centre of Investigation on Environment Genetics and Oncobiology (CIMAGO), Institute for Clinical and Biomedical Research (iCBR), Faculty of Medicine, University of Coimbra, 3004-531 Coimbra, Portugal; 5 Centre for Innovative Biomedicine and Biotechnology (CIBB), Group of Environment, Genetics and Oncobiology (CIMAGO)—Institute for Clinical and Biomedical Research (iCBR), Faculty of Medicine (FMUC), University of Coimbra, 3004-531 Coimbra, Portugal; 6 Laboratory of Oncobiology and Hematology, University Clinic of Hematology, Faculty of Medicine (FMUC), University of Coimbra, 3004-531 Coimbra, Portugal; 7 Health Human Movement Unit, North Health Polytechnic Institute, Cooperativa de Ensino Superior Politécnico e Universitário (CESPU), 4585-116 Gandra, Portugal; 8 Institute of Biomedicine (iBiMED), Department of Medical Sciences, University of Aveiro, 3810-193 Aveiro, Portugal; 9 Cardiology Department, Centro Hospitalar Universitário do Porto, Portugal & Unit for Multidisciplinary Investigation in Biomedicine (UMIB), Institute for Biomedical Sciences Abel Salazar, University of Porto, 4099-002 Porto, Portugal; 10 Laboratory for Integrative and Translational Research in Population Health (ITR), University of Porto, 4050-600 Porto, Portugal

**Keywords:** acute exercise, cardiovascular disease, endothelium, flow cytometry

## Abstract

The impact of exercise on the levels of endothelial progenitor cells (EPCs), a marker of endothelial repair and angiogenesis, and circulating endothelial cells (CECs), an indicator of endothelial damage, in heart failure patients is largely unknown. This study aims to evaluate the effects of a single exercise bout on the circulating levels of EPCs and CECs in heart failure patients. Thirteen patients with heart failure underwent a symptom-limited maximal cardiopulmonary exercise test to assess exercise capacity. Before and after exercise testing, blood samples were collected to quantify EPCs and CECs by flow cytometry. The circulating levels of both cells were also compared to the resting levels of 13 volunteers (age-matched group). The maximal exercise bout increased the levels of EPCs by 0.5% [95% Confidence Interval, 0.07 to 0.93%], from 4.2 × 10^−3^ ± 1.5 × 10^−3^% to 4.7 × 10^−3^ ± 1.8 × 10^−3^% (*p* = 0.02). No changes were observed in the levels of CECs. At baseline, HF patients presented reduced levels of EPCs compared to the age-matched group (*p* = 0.03), but the exercise bout enhanced circulating EPCs to a level comparable to the age-matched group (4.7 × 10^−3^ ± 1.8 × 10^−3^% vs. 5.4 × 10^−3^ ± 1.7 × 10^−3^%, respectively, *p* = 0.14). An acute bout of exercise improves the potential of endothelial repair and angiogenesis capacity by increasing the circulating levels of EPCs in patients with heart failure.

## 1. Introduction

Heart failure (HF) is a clinical syndrome characterized by structural and functional alterations in the cardiovascular system [1]. Patients with HF present abnormal hemodynamic alterations, such as increased intracardiac pressures and/or depressed cardiac output, which can be evidenced during physical efforts and/or at rest [1]. The endothelium plays a paramount role in hemodynamic control and vascular function [2,3]. Endothelial dysfunction is related to the progression of HF [4], being an independent predictor for HF [5] and a potential treatment target [6]. In a healthy endothelium, there is a delicate balance between endothelial injury (e.g., assessed by the levels of endothelial damage biomarkers, such as circulating endothelial cells (CECs)) and the endogen repair capacity (e.g., assessed by the circulating levels of pro-angiogenic and endothelial repair/maintenance factors, such as endothelial progenitor cells (EPCs)) [7].

The EPCs are bone-marrow-derived cells with the potential to migrate and differentiate in mature endothelial cells. EPCs are attracted to sites of endothelial damage, contributing to endothelial repair, maintenance, and angiogenesis [8]. Indeed, the EPCs can also be described through their biological properties and the time of growth in vivo culture, namely as early EPCs and late EPCs. Early EPCs are known to participate in the formation of vessels through paracrine mechanisms, while late EPCs participate in endothelial tubulogenesis [9]. The number of EPCs is positively related to endothelial function [10] and has been used for the assessment of endothelial dysfunction [4]. Functionality and levels of EPCs are reduced in HF patients [11,12] and tend to decline with aging [13]. Indeed, reduced circulating levels of EPCs are related to increasing hazard ratios for all-cause mortality and cardiovascular death, independently of N-terminal pro B-type natriuretic peptide (NT-proBNP) levels [14], and circulating levels of EPCs are considered a strong and independent predictor of mortality in HF [15,16].

The CECs are mature endothelial cells that have detached from the endothelial layer of the vessel after endothelial injury [17], being the most direct cellular marker of endothelial damage [18,19,20]. CECs are present at low levels in healthy subjects [21], whereas high levels are present in cardiovascular diseases [22], including HF [21,22]. Recently, CECs were proposed as a diagnostic biomarker for HF with preserved ejection fraction (HFpEF) [23], which highlights the potential of the CEC count for clinical settings.

Physical exercise can improve exercise capacity and decrease HF hospitalizations, being strongly recommended in the treatment of patients with HF [1]. One of the benefits of exercise training among patients with HF seems to be the increased mobilization of EPCs from bone marrow [24,25,26]. A growing number of studies have investigated the influence of isolated exercise sessions, namely maximal exercise, in the mobilization of EPCs in HF patients [27,28,29,30,31,32]. However, none considered the additional analysis of the effects of a maximal exercise bout on endothelial damage indicators. The optimization of strategies that could increase the endogenous mobilization of EPCs without inducing endothelial damage is of undeniable clinical importance. Thus, the present study aims to evaluate the impact of a maximal exercise bout on the levels of EPCs and CECs in HF patients. We hypothesized that a maximal exercise bout increases the levels of EPCs to those of age-matched individuals. Therefore, we assessed the circulating levels of EPCs and CECs in HF patients before and after a cardiopulmonary exercise test (CPET) and compared it with the resting circulating levels of age-matched adults free from cardiovascular disease.

## 2. Materials and Methods

### 2.1. Participants and Design of the Study

Thirteen patients with chronic HF were recruited from the cardiac rehabilitation program of the North Rehabilitation Center, Vila Nova de Gaia, Portugal. The eligibility was assessed by a clinician based on the following inclusion criteria: patients with HF according to the European Society of Cardiology (ESC) guidelines [1], age ≥ 45 years old; New York Heart Association (NYHA) class I–III; clinically stable. All patients were referred to the cardiac rehabilitation program after an HF diagnosis. In brief, the diagnosis was established after the assessment of clinical history, physical examination, brain natriuretic peptide evaluation, electrocardiogram, and echocardiography to confirm structural and/or functional alterations of the heart [1]. Participants with lung disease, peripheral artery disease, heart transplantation and/or exercise-limiting orthopedic disabilities were excluded. Additionally, a group of 13 age-matched adults was recruited to serve as a reference group for baseline EPC and CEC levels. The reference group was composed of adults with cardiovascular risk factors referred by their physicians for participation in a primary prevention program. The inclusion criteria were the presence of cardiovascular risk factors but free from cardiovascular disease; age ≥ 45 years old. The exclusion criteria were the same as for the HF group.

The clinical and sociodemographic data were retrieved from the clinical files and confirmed with the participants. Body weight, height, and the level of adherence to the Mediterranean Diet were assessed before the CPET. Adherence to the Mediterranean Diet was assessed with the 14-Item Mediterranean Diet Adherence Screener (MEDAS); the result of the MEDAS was obtained by summing the score (0 or 1) assigned to each question (0–14). A total score ≤ 5 indicates “weak adherence”, 6 to 9 “moderate to fair adherence,” and ≥10 “good or very good adherence” to the Mediterranean diet [33]. Blood collection was performed before and 30 min after the completion of the CPET to assess the number of EPCs and CECs.

All the participants provided written informed consent. The study procedures were in accordance with the Declaration of Helsinki and the study was approved by the Ethics Committee for Social and Health fields of the Santa Casa da Misericórdia do Porto (the entity that at that time was running the North Rehabilitation Center) (Ata No. 28, 28 March 2017).

### 2.2. Cardiopulmonary Exercise Test

Patients underwent a symptom-limited maximal CPET in the afternoon (between 2 and 4 pm), using a calibrated electronic treadmill (Bruce protocol). Patients were instructed to take all medications on the test day and were stimulated to exercise until exhaustion. Briefly, the cardiac rhythm was assessed by a 12-lead electrocardiogram throughout the CPET. Blood pressure was recorded at rest and during the CPET. Ventilation (VE), oxygen uptake (VO_2_), and carbon dioxide output (VCO_2_) were measured breath-by-breath using the CS-200 gas analyzer Ergo Spiro (Schiller, Baar, Switzerland). The VO_2_ peak (mL/kg/min) was considered the highest value reached at the end of the test, and the respiratory exchange ratio was registered for the evaluation of the level of effort. The VE/VCO_2_ slope was calculated by automatic linear regression fitting the relationship between VE and VCO_2_. The duration of the exercise test (min) was recorded. All the included HF patients completed the symptom-limited maximal CPET.

### 2.3. Quantification of Circulating Number of EPCs and CECs

Blood samples (3 mL) for cytometry analysis were collected into ethylenediaminetetraacetic acid (EDTA) tubes and treated, according to the manufacturer’s instructions, with TransFix (Cytomark, Caltag Medsystems Ltd., Buckingham, UK) at a 1:5 ratio immediately after collection. Transfix can stabilize cell populations and permits blood analysis for up to seven days after blood collection [34]. Blood samples were collected in the afternoon between 2 and 4 pm (patients were not fasting), before and 30 min after the CPET, and stored in the dark at room temperature. The flow cytometry analysis (FACS-Calibur flow cytometer, Becton Dickinson, San Jose, CA, USA) was performed two to three days after the blood collection. Staining and analysis were performed using a protocol adapted from Ahmed, et al. [35], as previously reported [11]. For the quantitative assessment of circulating EPCs and CECs by flow cytometry, whole blood samples were incubated for 10 min with FcR-blocking reagent to block unwanted binding of antibodies to human Fc receptor-expressing cells. All staining procedures were executed at room temperature. Samples were incubated with BV410 CD34 (BD Horizon, BD, Franklin Lakes, NJ, USA), PE CD309 (VEGFR-2/KDR; BD Pharmingen, BD, Franklin Lakes, NJ, USA), FITC CD144 (BD Pharmingen, BD, Franklin Lakes, NJ, USA), BV510 CD45 (BD Horizon, BD, Franklin Lakes, NJ, USA), and APC CD133/1 (Miltenyi Biotec, Cologne, Germany), according to manufacturer’s instructions. After erythrocyte lysis, at least 500,000 CD45^+^ and a minimum of 100 CD34^+^ cells were acquired on a BD FACS Canto II™ system using BD FACSDiva™ version 6.1.3 software (BD Biosciences, Franklin Lakes, NJ, USA). All samples were analyzed in duplicate. Data were analyzed using Infinicyt^TM^ (Cytognos, Salamanca, Spain). The EPCs were defined as CD45^low^/CD34^+^/CD309^+^/CD133^+^/CD144^−^ cells [36] and the CECs as CD45^low^/CD34^+^/CD309^+^/CD133^−^/CD144^+^ cells [7,23,37]. EPC and CEC counts were expressed as % leukocytes (CD45^+^ cells). The intra-assay variation was <5%.

### 2.4. Statistical Analysis

Exploratory analysis and Shapiro-Wilk tests were performed to determine the normality of the data distribution. Variables are expressed as mean ± standard deviation, mean differences with their 2-sided 95% CIs or absolute number and percentage. Paired-sample T-tests were performed for the within-group comparisons (EPCs and CECs) from baseline to 30 min after the CPET. Between-group comparisons were performed using the Student’s independent *t*-test (EPCs, CECs, and clinical characteristics), Chi-squared (χ2) test, or Fisher’s exact test (clinical characteristics). Also, the HF patients were divided into two groups according to the severity of exercise intolerance (based on the median VO_2_ peak: 17.0 mL/kg/min); then, a student’s independent *t*-test was performed to assess whether those with lower cardiorespiratory fitness show a similar EPC response to those with higher fitness. The value of significance was set at a 1-sided *p* value ≤ 0.05. Data analyses were made using IBM SPSS Statistics 26 (IBM Corp., Armonk, NY, USA).

## 3. Results

### 3.1. Participants’ Characteristics

HF patients and age-matched adults free from cardiovascular disease were well balanced for age (67.8 ± 9.7 vs. 65.7 ± 7.1 years old, *p* = 0.52) and body mass index (27.7 ± 3.6 vs. 29.6 ± 5.6 kg/m^2^, *p* = 0.30) (Table 1). Some cardiovascular risk factors were more prevalent in HF patients, including diabetes mellitus (53.8%) and hypertension (84.6%). The HF patients showed a reduced left ventricular ejection fraction (LVEF) (37.2 ± 12.0%) and VO_2_ peak (16.8 ± 4.1 mL/kg/min; 76.2 ± 22.9% of predicted). The VE/VCO_2_ slope was 34.6 ± 10.9 and the duration of the CPET was 9.0 ± 1.9 min. All patients completed the maximal exercise bout without signs or symptoms of ischemia. At baseline, HF patients presented a significantly lower number of circulating EPCs (*p* = 0.03) but similar levels of CECs (*p* = 0.08) when compared to the age-matched group (Table 1).

### 3.2. Effects of Maximal Exercise in the Levels of EPCs and CECs

**Figure 1:**
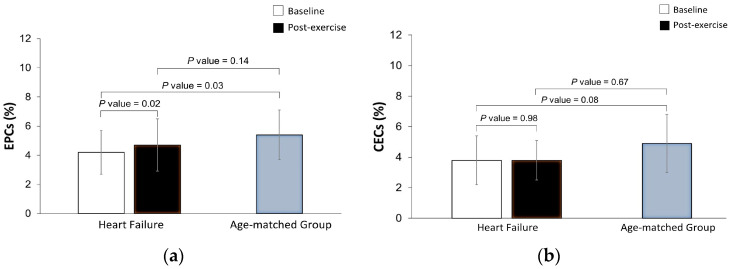
Effects of a maximal exercise bout on the circulating levels of endothelial progenitor cells (EPCs, Panel (**a**)) and circulating endothelial cells (CECs, Panel (**b**)) in heart failure patients, and comparison between baseline and post-exercise bout levels in the HF group with the resting circulating values of age-matched adults free from cardiovascular disease. EPCs and CECs count were expressed as % leukocytes (% of circulating cells × 10^−3^).

In HF patients, a single maximal exercise bout increased the levels of EPCs by 0.5 [95% Confidence Interval (CI), 0.07 to 0.93%], i.e., from 4.2 × 10^−3^ ± 1.5 × 10^−3^% to 4.7 × 10^−3^ ± 1.8 × 10^−3^% (*p* = 0.02) (Figure 1a). The circulating levels of CECs did not change in response to the exercise bout (3.8 × 10^−3^ ± 1.6 × 10^−3^% to 3.8 × 10^−3^ ± 1.3 × 10^−3^%, *p* = 0.98) (Figure 1b). The EPC response to the exercise bout was similar among HF patients with lower (<17.0 mL/kg/min) versus higher VO_2_ peak (≥17.0 mL/kg/min) (0.41 ± 0.77 vs. 0.69 ± 0.71%, *p* = 0.53).

When comparing the levels of EPCs of the HF patients after the maximal exercise bout with the resting value of the age-matched group, we observed that the circulating level of EPCs in HF patients was not significantly different from the level of the age-matched participants (4.7 × 10^−3^ ± 1.8 × 10^−3^% vs. 5.4 × 10^−3^ ± 1.7 × 10^−3^%, respectively, *p* = 0.14) (Figure 1a).

## 4. Discussion

The main findings of this study confirm our hypothesis that a maximal exercise bout increases the circulating levels of EPCs in HF patients, and that the number of EPCs in circulation after the exercise bout is similar to the baseline count of age-matched adults free from cardiovascular disease. Moreover, acute exercise improves EPC levels without increasing endothelial damage, assessed by measuring the levels of CECs.

The effects of acute exercise on EPCs are still uncertain, with previous studies reporting increased levels of EPCs after a maximal exercise test [27,28,29] while others describing no differences [30,31,32,38,39]. For instance, Kourek, et al. [27] observed an increase in the levels of EPCs after a maximal exercise bout performed on a cycle ergometer. The same authors tested the influence of HF severity on the ability to mobilize EPCs after a maximal exercise and concluded that the ability to mobilize EPCs after a maximal exercise was not affected by HF severity, regardless of the criteria (VO_2_ peak, predicted VO_2_ peak or VE/VCO_2_) used to determine HF severity [28]. Another study found a nearly four-fold increase in the number of EPCs [40]. Our preliminary results tend to indicate that the EPC response to a maximal exercise bout is similar irrespective of patients’ VO_2_ peak. On the other hand, Van Craenenbroeck, et al. [31] did not find significant changes in EPCs at several time points after a maximal exercise test (i.e., 10 min, 30 min, 1 h, 2 h, 4 h, 8 h, 12 h, 24 h, 48 h) in a group of patients with chronic HF. Some authors suggested that the blunted response of EPCs to exercise may be related to inflammatory factors and diminished availability of nitric oxide (NO) in response to exercise [41,42]. It must be highlighted that the literature is not consistent in the antibodies used to define or select cells that express properties attributed to EPCs, for instance, EPCs have been identified as CD34^+^/CD133^+^/VEGFR_2_, CD34^+^/CD45^−^/CD133^+^/VEGFR_2,_ and CD34^+^/CD45^−^/CD133^+^ [27,28,29] or CD34^+^/KDR^+^/CD3^−^ [30,31,32] or CD34^+^/KDR^+^ [38], CD34^+^/KDR^+^/CD45^dim^ [39], and CD133^+^/CD144^+^ (or AC133^+^/VE-Cadherin^+^) cells [40]. Additionally, the different units used to count circulating EPCs (e.g., cells per event, percentage of cells within the lymphocyte or the total mononuclear cell population, or cells per volume) make comparisons between studies difficult.

The exercise characteristics (e.g., intensity and duration) must be considered when evaluating the physiological response of endothelial repair mechanisms to exercise. The intensity and type of exercise may have a bigger influence on EPC dynamics than the duration. Laufs and colleagues tested the acute effects of different durations of exercise and found that a 10-min aerobic exercise session at moderate intensity does not change the levels of EPCs, while a 30-min exercise session at moderate to vigorous intensity increases EPCs in healthy young men [43]. Moreover, a recent study tested chronic HF patients (EF ≤ 45%) and found that both 31 min of high-intensity interval exercise—4 min of aerobic exercise at 80% VO_2_ peak intermitted by 3 min of active recovery at moderate intensity (50% VO_2_ peak)—and 53-min of continuous exercise at moderate intensity (50% VO_2_ peak) (both exercise sessions were similar in total work) increased the levels of EPCs immediately and 40 min after the end of the exercise sessions [44]. In this sense, considering that in the present study, the exercise bout lasted an average of 9 min, and CPET is characterized by an effort of short duration and high intensity [45], we may assume that the intensity of exercise may be more important to the mobilization of EPCs than the duration of exercise.

Our results showed lower levels of EPCs in patients with HF at rest, which is in line with previous research [11,16,46]. Indeed, HF patients may present a down-regulation of endothelial nitric oxide synthase and increased oxidative stress that may affect the mobilization of EPCs [4,14]. These mechanisms participate in the progression of endothelial dysfunction and contribute to a lower capacity of endothelial repair and regeneration in HF [4,14].

**Figure 2:**
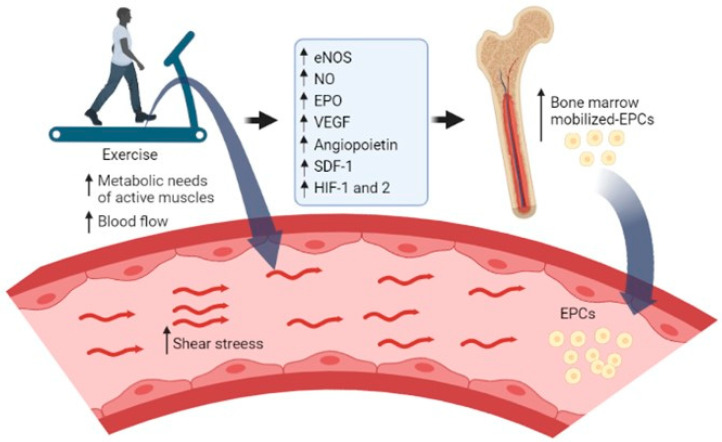
Summary of the potential factors involved in endothelial progenitor cell (EPC) mobilization during exercise. During exercise, the increase (↑) in cardiac output and blood flow to active muscles increase the exercise-induced shear stress that stimulates the endothelial cells to increase the activity of endothelial nitric oxide synthase (eNOS) and produce higher amounts of nitric oxide (NO) [42]. The increase in NO [42,47,48], hypoxia-inducible factor (HIF)-1 and -2, erythropoietin (EPO) [25], and other angiogenic growth factors or chemokines, namely the vascular endothelial growth factor (VEGF), angiopoietin and stromal cell-derived factor (SDF)-1 bonding with the chemokine receptor (CXCR-4) [49,50] seem to mediate the mobilization of EPCs from bone marrow to peripheral blood [25]. The figure was designed by using the BioRender.com resource.

The present study did not assess the mechanisms by which exercise would increase the number of EPCs in HF patients. However, a schematic diagram summarizing the potential pathways involved in EPCs dynamics is provided in Figure 2.

Regarding the levels of CECs, we did not find significant differences in response to maximal exercise in HF patients. Previous studies showed that a maximal exercise session [51] or 30 min of high-intensity interval exercise [52] did not change the CEC count in healthy young men. However, a previous study reported an increase in CEC levels immediately after a CPET, which was followed by a significant fall 30 min after exercise among coronary artery disease patients [19]. This biphasic pattern was also observed in 13 patients with effort angina (i.e., patients diagnosed during the exercise, which triggered angina pectoris or ST segment depression during the CPET) since the CEC count increased after exercise testing, followed by a decrease 4 h after the exercise test [53]. An acute exercise session [54] can lead to a biphasic acute response in endothelial function, i.e., a decrease in endothelial function followed by a significant improvement until reaching or overcoming the resting levels of endothelial function [54,55]. As recently discussed [54], acute exercise at high intensities can lead to endothelial damage, which would be reflected in decreased endothelial function and increased circulating indicators of endothelial damage (e.g., CECs). Thus, the activation of vascular repair mechanisms (e.g., mobilization of EPCs) would increase the repair capacity of the endothelium. Therefore, the endothelial damage could be detectable only for a short period, i.e., until vascular repair mechanisms act to repair the endothelial damage [54]. Methodological differences, such as the lack of standardization of surface markers for the quantification of CECs, limit the comparisons between studies and may, at least partially, explain divergent results.

Our study presented some limitations. Firstly, we only collected blood at one time point after the CPET. Secondly, the present study lacks additional analysis of other endothelial damage markers (e.g., endothelial-derived microparticles [7] and inflammatory cytokines [41], which could provide further information regarding the effects of maximal exercise on endothelial damage. Finally, age-matched subjects did not perform a CPET, which limits the comparison of the exercise-induced EPC response between them and HF patients. Despite these limitations, these preliminary results may provide new insights and perspectives into the effects of an acute exercise session on vascular health among HF patients. Future research with a larger sample and evaluating additional indicators of endothelial repair and vascular angiogenesis is necessary to confirm our findings.

## 5. Conclusions

A maximal exercise bout seems to increase the circulating levels of EPCs in HF patients without increasing endothelial damage (CEC count) in HF patients.

## Figures and Tables

**Table 1:** Clinical characteristics of the participants.

	Heart Failure Group (n = 13)	Age-Matched Group (n = 13)	*p*
Sex (males/females)	13 (11/2)	13 (2/11)	≤0.001
Age (years)	67.8 ± 9.7	65.7 ± 7.1	0.52
Anthropometric data			
Weight (kg)	75.6 ± 11.9	73.2 ± 16.7	0.68
Height (cm)	165.1 ± 9.6	156.7 ± 10	0.03
Body mass index (kg/m^2^)	27.7 ± 3.6	29.6 ± 5.6	0.30
Cardiovascular risk factors, n (%)			
Overweight	5 (38.5)	6 (46.2)	0.69
Obesity	4 (30.8)	5 (38.5)	1.00
Hypertension	11 (84.6)	7 (53.8)	0.20
Diabetes mellitus	7 (53.8)	4 (30.8)	0.23
Dyslipidaemia	8 (61.5)	8 (61.5)	1.00
Heart failure characteristics			
HFrEF, n (%)	8 (61.5)	-	-
HFmrEF, n (%)	4 (30.8)	-	-
HFpEF, n (%)	1 (7.7)	-	-
LVEF, %	37.2 ± 12.0	-	-
NYHA functional class, n (%)			
I	2 (15.4)	-	-
II	11 (84.6)	-	-
Aetiology			
Ischemic, n (%)	7 (53.8)	-	-
Dilated, n (%)	6 (46.2)	-	-
Current medication, n (%)			
Statins	10 (76.9)	8 (61.5)	0.67
Antidiabetic medication	6 (46.2)	3 (23.1)	0.41
Diuretics	9 (69.2)	5 (38.5)	0.11
Angiotensin II receptor blockers	1 (7.7)	2 (15.4)	1.00
Angiotensin converting enzyme inhibitors	10 (76.9)	3 (23.1)	0.006
Calcium channel blockers	2 (15.4)	1 (7.7)	1.00
Beta-blockers	8 (61.5)	0 (0.0)	0.002
Mediterranean Diet Adherence			
Total scoring	8 ± 2	8 ± 2	0.47
Weak adherence, n (%)	0 (0.0)	1 (7.7)	1.00
Moderate to fair adherence, n (%)	11 (84.6)	8 (61.5)	0.37
Good or very good adherence, n (%)	2 (15.4)	4 (30.8)	0.64
Circulating Cells Populations			
Endothelial progenitor cells (%)	4.2 × 10^−3^ ± 1.5 × 10^−3^	5.4 × 10^−3^ ± 1.7 × 10^−3^	0.03
Endothelial cells (%)	3.8 × 10^−3^ ± 1.6 × 10^−3^	4.9 × 10^−3^ ± 1.9 × 10^−3^	0.08

Legend: HFrEF, heart failure with reduced ejection fraction; HFmrEF, heart failure with mid-range ejection fraction; HFpEF, heart failure with preserved ejection fraction; LVEF, left ventricular ejection fraction; NYHA, New York Heart Association.

## Data Availability

Deidentified data presented in this study will be made available for researchers who provide a methodologically sound proposal (e.g., individual participant meta-analysis).
